# Mapping Chemical-Gene Interactions for Developmental Lethality and Pregnancy Loss

**DOI:** 10.1101/2025.10.03.25337209

**Published:** 2025-10-07

**Authors:** Syed Hassan Bukhari, Amrita Nagasuri, Boris Oskotsky, Leen Arnaout, Polina Minkovski, Gonçalo D.S. Correia, David A. MacIntyre, Gary M. Shaw, David K. Stevenson, Ruth B. Lathi, Svetlana A. Yatsenko, Tomiko T. Oskotsky, Aleksandar Rajkovic, Marina Sirota

**Affiliations:** 1 Bakar Computational Health Sciences Institute, University of California, San Francisco, CA, USA; 2 Parturition Research Group, Institute of Reproductive & Developmental Biology, Department of Metabolism, Digestion and Reproduction, Imperial College London, London, UK; 3 March of Dimes Prematurity Research Centre at Imperial College London, London, UK; 4 Robinson Research Institute, University of Adelaide, Australia; 5 Department of Pediatrics, Stanford University, Stanford, CA, USA; 6 Department of Obstetrics and Gynecology, Stanford University, Stanford, CA, USA; 7 Department of Pathology, Stanford University, Stanford, CA, USA; 8 Division of Clinical Informatics and Digital Transformation, Department of Medicine, University of California, San Francisco, San Francisco, CA, USA; 9 Department of Pathology, University of California, San Francisco, San Francisco, California CA, USA; 10 Institute of Human Genetics, University of California, San Francisco, San Francisco, CA, USA; 11 Department of Pediatrics, University of California, San Francisco, San Francisco, CA, USA

**Keywords:** Pregnancy Loss, Recurrent Pregnancy Loss (RPL), Chemical-Gene Interactions, Developmental Lethality, Exposomics

## Abstract

**Purpose:**

Pregnancy loss affects 10–15% of clinically recognized pregnancies and is driven by genetic and environmental factors. Interactions between genes and chemicals critically shape developmental outcomes, yet existing resources do not organize chemical-gene evidence by gestational timing, maternal-fetal compartment, or lethality context.

**Methods:**

To minimize this gap, we merged Intolerome genes and the Comparative Toxicogenomics Database databases to create a filtered network of 928 lethality-associated genes and ~4,000 chemicals. We developed the Chemical-Gene Atlas (CGA) application, which analyzes chemical-gene interactions using four filters supporting hypothesis generation and clinical translation.

**Results:**

Using recurrent pregnancy loss (RPL) as a case study, CGA identified five clinically important genes (F5, F2, AURKB, PADI6, and FOXD1) demonstrating different exposomic patterns with Bisphenol A (BPA) and Benzo[a]pyrene (B[a]P). These exposures affect gene expression, methylation, and coagulation pathways and placental function. Our findings demonstrate that genes with loss-of-function intolerance, especially those expressed in metabolic and cardiovascular systems, are susceptible to environmental disruption during critical windows of development, including implantation, placental development, and early organogenesis.

**Conclusion:**

The CGA platform provides a scalable model to investigate chemical-gene interactions leading to developmental lethality. CGA is available at https://cgatlas.org/.

## Introduction

Pregnancy loss, specifically miscarriage before 20 weeks of gestation, affects 10–15% of all clinically recognized pregnancies, and rates are much higher if preclinical and biochemical pregnancy losses are included (Lathi et al., 2014.. Recurrent pregnancy loss (RPL), defined as two or more pregnancy losses, affects ~5% of couples (Quintero-Ronderos et al., 2019). RPL takes an emotional and psychological toll on families and individuals, leading to anxiety and uncertainty about future pregnancies (Quenby et al., 2021). Understanding the genetic etiology and environmental influences of RPL can inform family planning and targeted interventions, as research increasingly demonstrates that these factors significantly impact embryonic survival and pregnancy outcomes (Puche-Juarez et al., 2023).

Environmental exposures further compound this risk. Endocrine-disrupting chemicals (EDCs) interfere with the body’s hormonal balance and can impact fertility and pregnancy outcomes (Puche-Juarez et al., 2023). Heavy metals such as lead, mercury, and cadmium can also result in fetal growth restriction, neurodevelopmental complications, and placental insufficiency (Zinia et al., 2023). Air pollutants, such as particulate matter, nitrogen dioxide, and carbon monoxide, can lead to reduced placental blood flow, inflammatory and oxidative stress responses that damage fetal DNA, and increase the risk of preterm birth or even stillbirth (Fussell et al., 2023). These well-established links between environmental exposures and reproductive outcomes illustrate why improved integrative tools are needed.

However, current toxicogenomic resources are poorly aligned with pregnancy-loss decision-making. They typically lack annotations for gestational timing, maternal-fetal compartment, and lethality, and they do not systematically prioritize high-risk developmental genes. As a result, clinicians and researchers cannot readily ask which exposures matter, when in gestation they matter, and in which compartment they may act to cause pregnancy loss.

For instance, the Intolerome database catalogs 934 genes essential for fetal viability, most of which reflect fetal genetic contributions (Intolerome, 2025). The Comparative Toxicogenomics Database (CTD) offers a free, peer-reviewed resource that contains meticulously curated information on chemical-gene-disease interactions (Davis et al., 2025). While valuable, CTD does not allow exploration of chemical–gene interactions by developmental stage, affected biological systems, or lethality phases, thereby limiting its relevance for reproductive toxicology and RPL research. Its output remains largely static and tabular, restricting deeper network-level analysis.

To address this gap, we developed the Chemical-Gene Atlas (CGA), an interactive, publicly accessible resource that integrates loss-of-function intolerant genes data found in the Intolerome with curated chemical-gene interaction data found in the Comparative Toxicogenomics Database (CTD). The CGA enables the user to filter and visualize close to ~900 genes and ~4,000 chemicals by developmental timing, biological system, gene effect, and functional mechanism, providing a novel means of mapping the interaction between environmental exposures and genetic vulnerability. CGA represents the first effort to systematically unify intolerance, exposure, and developmental context into an interactive platform for pregnancy-loss research. In this framework, an “interaction” is an environmentally induced perturbation mapped onto a loss-of-function–intolerant gene, contextualized by gene effect, developmental timing, system affected, and lethality mode.

Gene-Environment interactions (G × E), including chemical-gene interactions, are central to developmental disease risk, but current resources like the CTD lack integrative tools that combine gene intolerance, chemical exposure, and developmental timing. Our approach integrates genetic intolerance with exposomic data to reveal patterns of chemical–gene interactions unique to developmental outcomes. We hypothesized that this framework would help prioritize vulnerable genes and exposure risks. As a proof of concept, we applied it to recurrent pregnancy loss (RPL) to explore how environmental exposures interact with fetal gene networks.

## Methods

CGA utilizes a pre-merged dataset made from two public datasets: Intolerome, which provides data on genetic intolerance and gene-disease association (links between genetic variants and elevated risk of clinical outcomes such as developmental disorders or pregnancy loss), and CTD (Comparative Toxicogenomics Database), containing curated chemical-gene interactions (evidence that chemical exposures alter gene expression, activity, or function) and disease associations (Davis et al., 2025; Intolerome, 2025). The merged dataset matches genes based on OMIM Gene ID from Intolerome to CTD, filtering for the chemical-gene interactions explicitly focused on prenatal and postnatal disease phenotypes, genetic lethality, and developmental disorders.

### Data Cleaning

The dataset underwent multiple preprocessing and cleaning steps before and after the merge. Before the merge, redundant white spaces, symbols, and non-informative placeholders were removed to create a more consistent and uniform dataset. Columns were restructured to ensure consistency between the pre- and post-merged datasets. Furthermore, the lethality timing data were reshaped from a wide format into a long format for flexible downstream filtering and visualization. Finally, duplicates for the same chemical-gene interactions were removed by retaining distinct rows based on key identifiers (e.g., gene and chemical names). This was done to ensure clean network plots, reduce redundancy, and improve relevance.

### User Interface

We utilized CSS and HTML within R Shiny to structure and style the user interface for the web application. Dynamic filter controls were created to ensure a smooth filtering process. Four main filter dropdown menus are presented:

1. Gene Effect:
  Gene effect categorizes genes according to their role in developmental stages, particularly fetal and neonatal outcomes. In this context, effect refers to the degree to which variation in a gene contributes to measurable risks, such as growth restriction, congenital malformations, or prenatal/neonatal lethality. By capturing these associations, the framework highlights how genetic variation influences developmental phenotypes and organizes genes into four categories: fetal, maternal, maternal–fetal, and unknown.
2. System Affected:
  This category organizes genes based on the biological systems they impact, such as cardiovascular, nervous, or metabolic systems. Therefore, it provides an opportunity for targeted investigation of system-specific disorders of disease mechanisms and pathways of disrupted genetic alterations.
3. Lethality Mode of Function:
  Lethality Mode of Function differentiates gene variants by their functional impact, mainly around loss-of-function or gain-of-function variants, with rare cases of duplication and deletion in our dataset. This distinction enables researchers to focus on genes that drive lethality and developmental anomalies, thereby devising therapeutic strategies and interventions.
4. Lethality Timing:
  Lethality Timing categorizes gene impacts according to the developmental timing of lethality, spanning various trimesters until the postnatal period. This temporal categorization will allow researchers to target specific windows to their disease of interest.

These filters dynamically update downstream visualizations and tables. Any action taken by the users undergoes reactive selections implemented through Shiny’s reactive programming to ensure connectivity between different app components.

### Donut Chart Visualizations

The four filters are implemented using interactive donut charts to summarize the distributions of each filter category. These plots are also reactive upon user selection, and each click on a segment updates the network plots, diving deeper and narrower into the interactions between genes and chemicals.

### Network Plots

The second component of this app is a visualization of the chemical-gene interactions using the visNetwork package (Almende B.V. & Thieurmel, 2022). Red nodes represent genes, green nodes represent chemicals, and edges represent the interaction. Node sizes scale proportionally to node degree (i.e., the number of connections), allowing for the prioritization of highly interconnected genes. We have included interactive features like hover effects, nearest neighbor highlighting, node selection via dropdown, and smooth transitions to explore different pathways and focus on specific genes or chemicals under each filter.

### Filtered Data Table

The last component of the app is a filtered table that includes global and column-specific filtering functionality. Hyperlinks to various other datasets, including CTD, NCBI Gene, OMIM, and PubMed, are provided, and the dataset is available on the app with easy export options.

### Reactive Logic and Performance Optimization

Given the size of the dataset and the number of chemical-gene interactions, reactive programming principles ensure responsive and accurate data representation based on user inputs. Apart from the reactive filters mentioned above, individual plotting and data rendering functions were encapsulated into reactive expressions, ensuring consistent data synchronization across all visual components and tables.

Additionally, we took additional steps to improve performance, considering the computational complexity. We leveraged asynchronous rendering with the future and promise libraries to manage computationally expensive tasks like network plotting without creating interruptions (Bengtsson, 2025; Cheng, 2025). Visual feedback in the form of loader indicators was provided during intensive calculations to improve the user experience. Lastly, we increased the maximum allowable global memory size to 600 MB while running multisession in R to prevent memory-limit errors during parallel processing.

### Deployment and Accessibility

The CGA app was developed and extensively tested in an R environment (version 4.3.2). Complete source code and comprehensive documentation are publicly accessible through GitHub, providing reproducibility, transparency, and encouraging community-driven enhancements: https://github.com/SyedHassan20/cga-chem-gene-atlas.

### Reactome pathway-enrichment workflow

For each toxicant, we filtered the interaction table for BPA or B[a]P, converted the resulting non-redundant gene symbols to Entrez IDs with bitr (org.Hs.eg.db), and ran enrichPathway (ReactomePA/clusterProfiler v4.8.3) against the default human Reactome universe using a one-tailed hypergeometric test; Benjamini-Hochberg-adjusted *p-value* < 0.10 (Wu et al., 2021; Yu & He, 2016). This p-value is chosen to retain power for small, exploratory gene sets while remaining stricter than the package’s 20 % default, which was considered significant.

## Results

### Study design

**Figure 1.**
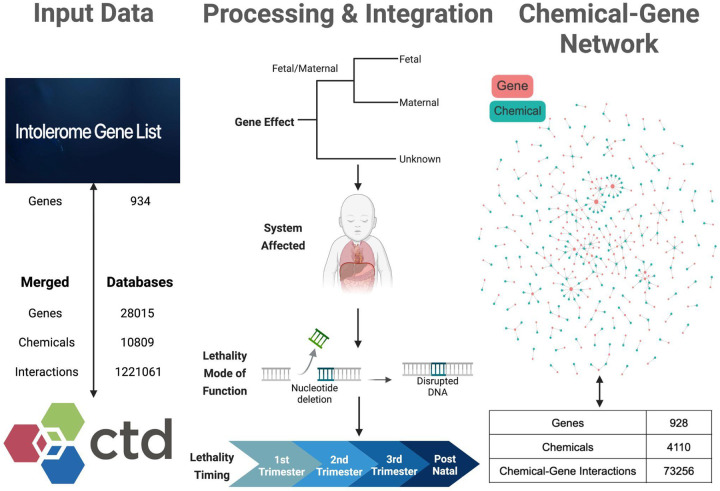
Integration of Intolerome Genes with Chemical-Gene Interaction Data to Construct a Chemical-Gene Network. Input Data is an initial list of 934 Intolerome genes that was filtered by Homo Sapiens and merged with CTD (Comparative Toxicogenomics Database) records containing 28,015 genes, 10,809 unique chemicals, and 1,221,061 chemical-gene interactions. Processing and Integration of the system provides annotations for fetal or maternal gene effects and unknown effects with specific lethal mechanisms, such as DNA-disruptive nucleotide deletions, and developmental period information ranging from the first trimester into postnatal stages. The Chemical-Gene Network is a chemical-gene interaction network constructed, comprising 928 genes and 4,110 chemicals, yielding 73,256 unique chemical-gene interactions. This network provides a framework to investigate the impact of environmental exposures on vulnerable developmental windows through chemical-gene interactions.

Our study integrated two large-scale datasets, Intolerome and CTD, to build an exposomic resource focused on chemical-gene interactions associated with developmental lethality (Figure 1). As summarized in Figure 1, the Intolerome dataset comprised 934 genes categorized as prenatal and postnatal genetic intolerance, which were used to identify suitable chemicals. Subsequently, the interactions involving these 934 genes in CTD focused on Homo sapiens. This merge reduced the ~10,000 unique chemicals and about 1.2 million chemical-gene interactions in CTD to 928 genes, ~4000 unique chemicals, and ~73000 total interactions (Figure 1). By restricting CTD interactions to those involving only the 928 intolerome genes in Homo sapiens, we provide a robust and interpretable source for exposomic network analysis, pathway enrichment, and the identification of vulnerable genes for various chemical exposures.

### Distribution of Gene Effects, Systemic Impacts, and Lethality Timing

**Figure 2.**
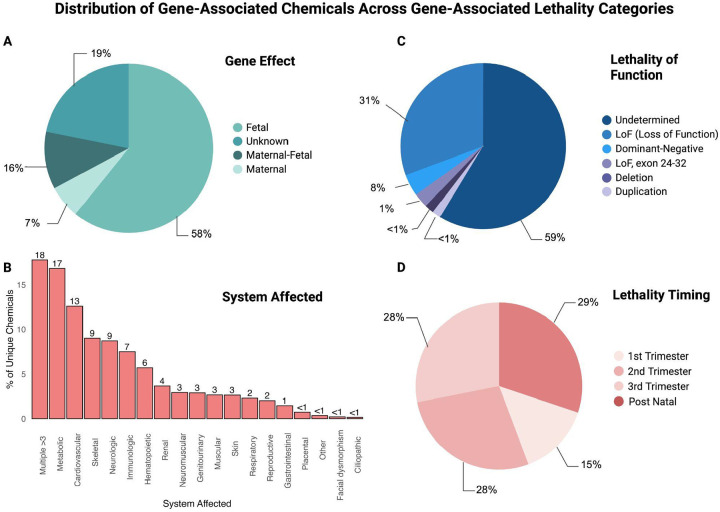
Distribution of Gene-Associated Chemicals Across Gene-Associated Lethality Categories. (A) Gene effect: most chemicals are associated with fetal-specific effects, with smaller subsets linked to maternal, maternal–fetal, or unknown origins in the merged dataset. **(B)** Physiological systems affected: chemicals most frequently impact the metabolic and multi-system categories, followed by cardiovascular and neurological systems. Less frequent associations occur in skeletal, immunologic, renal, and other systems. **(C)** Lethality mode of function: most associations are undetermined, with a substantial subset classified as loss-of-function (LoF). Additional categories include dominant-negative, LoF (exon 24–32), deletions, and duplications. **(D)** Lethality timing: chemicals are distributed across all trimesters, with a slightly greater contribution in postnatal lethality. Entries without defined lethality categories were excluded from this visualization.

After merging the dataset, we characterized the developmental relevance of the 928 genes in the merged dataset across four biological categories: gene effect (fetal vs maternal), affected physiological systems, lethality mode of function (loss of function, gain of function, etc), and timing of lethality (Figure 2). The merged dataset was used to build an interactive tool, CGA, available as an R Shiny application “cgatlas” (https://cgatlas.org/).

As shown in Figure 2A, 58% of unique chemicals are associated with fetal-specific effects, directly impacting embryonic or fetal development. Approximately 16% are linked to both maternal and fetal physiology. About 7% are associated with maternal-only effects, while 19% might have an unknown effect.

The distribution of genes across affected biological systems is shown in Figure 2B. The multi-systemic disorders represented the highest with 18% unique chemicals, followed by the metabolic system (17% unique chemicals) and the cardiovascular system (13%). Other systems with notable involvement were neurologic (9%), skeletal (9%), and immunologic (7%).

As shown in Figure 2C, most gene disruptions had an undetermined functional category. Among the classified variants, LoF events were the most prevalent, followed by dominant-negative effects, exon 24–32 specific LoF (FBN1), and structural variants such as deletions and duplications.

Lastly, Figure 2D shows the distribution of unique chemicals associated with lethality events across developmental timepoints: unique chemicals are most frequently implicated in postnatal outcomes (~29%), followed by the second (~28%), third (~28%), and first (~15%) trimesters.

### Chemical-Gene Interaction of RPL

The applicability of this application can be best described by providing an example of a disease and mapping its chemical-gene interactions. Therefore, we have extracted focused subnetworks of five integral genes (*F5, F2, AURKB, PADI6*, and *FOXD1*) associated with recurrent pregnancy loss (RPL), selected for their involvement in embryonic development, fertility, and survival during early life stages (Blengini & Schindler, 2024; Quintero-Ronderos et al., 2019; Sayal & Beksac, 2024; Zhou et al., 2024).

**Figure 3.**
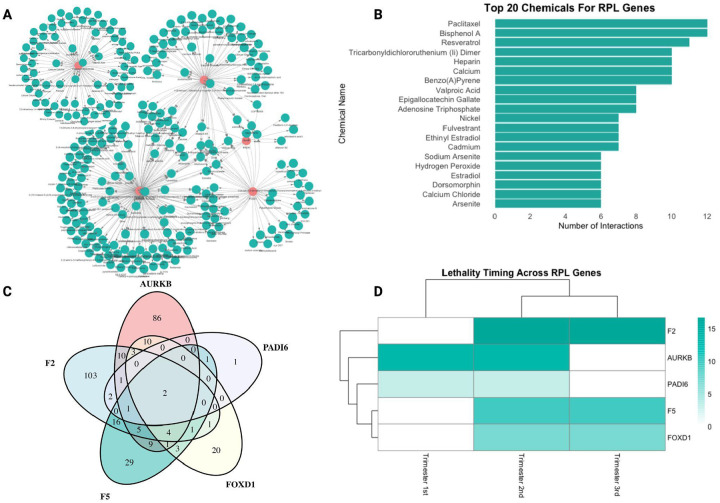
Analysis of Recurrent Pregnancy Loss (RPL) Genes, Chemicals, and Interactions. **(A)** Network visualization representing interactions between RPL genes (red nodes) and associated chemicals (green nodes), illustrating the extensive connectivity across all five genes. **(B**) Bar plot showing the top 20 chemicals associated with RPL genes, ranked by the number of interactions. Paclitaxel, bisphenol A, and resveratrol are among the most frequently linked chemicals. **(C)** Venn diagram displaying the overlap of chemicals across five key RPL-related genes (*AURKB, PADI6, F2, F5,* and *FOXD1*), which showcases the unique and common chemical-gene interactions between RPL genes. The center shows two main chemicals (Benzo(a)pyrene and bisphenol A) that interact with all five genes. **(D)** Heatmap representing lethality timing across different trimesters for selected RPL genes represented with a log scale, indicating variations in critical time windows for gene-associated lethality risks.

Each RPL gene was mapped with its full exposomic profile, visualized as networks in Figure 3A. A chemical was considered “linked” to a gene if a curated interaction between the two was present in CTD (e.g., documented effects on expression, activity, or binding, restricted to Homo sapiens). Node degree, defined as the number of unique chemical exposures interacting with a given gene, serves here as a quantitative proxy for environmental connectivity or sensitivity. Using this measure, F5 and AURKB genes exhibited the highest node degrees, whereas PADI6 showed a compact network with a narrower exposomic footprint. Additional information is provided in Figure 3D on the timing of lethality events, which shows that none of these genes affect postnatal outcomes, but all are implicated in the second trimester.

The Venn diagram and network plots were complemented by showing the top 20 most frequently associated chemicals for RPL in Figure 3B. These chemicals are ranked by their number of interactions across the five selected RPL genes to provide a high-level overview of prevalent exposures within the RPL gene set. This figure reflects Bisphenol A and Benzo(a)pyrene, the two chemicals common among all five genes, as having the second and sixth highest number of interactions with RPL genes.

We further focused our analysis by comparing shared versus unique environmental risks across all the RPL genes, as shown in Figure 3C. This Venn diagram represents the overlap in chemical interactors. Although most genes displayed distinct interaction profiles, Bisphenol A and Benzo(a)pyrene stood out, as they interact with all five RPL genes. In addition to overlap counts, each gene–exposure pair can be annotated with contextual features, such as node degree (overall environmental connectivity of the gene), the number of curated CTD interactions supporting that chemical-gene link, and the developmental timing of lethality, providing a proxy for “risk level” that may be more informative to clinicians considering environmental contributions to RPL.

**Figure 4.**
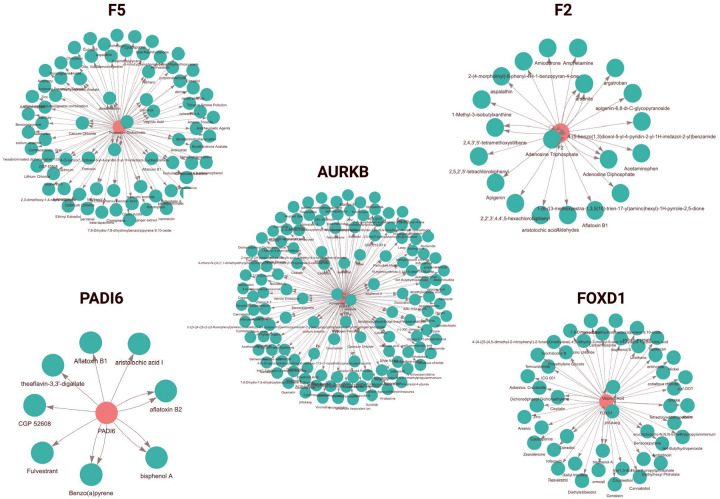
Exposomic Networks for Chemical-Gene Interactions of RPL Genes. Exposomics interaction networks show chemical associations for five genes related to RPL in genetic lethality and dysfunction. Each exposome network is centered around a gene (red node) and its chemical interactors (green nodes) derived from the Comparative Toxicogenomics Database (CTD). The networks feature varying degrees of environmental exposure susceptibility. *F5* and *AURKB* exhibit extensive chemical interactions, indicating broad environmental sensitivity, whereas *PADI6* demonstrates limited interactions, suggesting a more specialized exposome profile. *F2* and *FOXD1* networks display intermediate connectivity. The collection of these exposome profiles relates to chemical exposures and genetic expression, disease progression, and developmental outcomes.

Extending upon our exploration of the interactions between RPL genes and associated chemicals in Figure 3A, we have isolated the exposomic networks for the five RPL genes, shown in Figure 4. The size and density of each network reflect the relative exposure burden. The genes *F5* and *AURKB* have the highest number of connections and broad exposomic profiles.

In contrast, *PADI6* displays a smaller, more focused network. Lastly, *FOXD1* and *F2* fall in between, with a moderate number of chemical connections.

**Figure 5.**
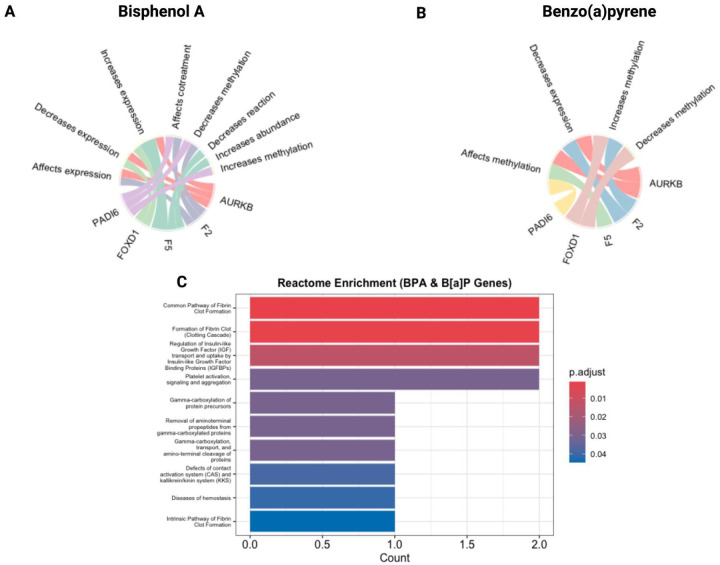
Chord diagrams and Reactome pathway enrichment for Bisphenol A (BPA) and Benzo[a]pyrene (B[a]P). Chord diagrams (A, B) show chemical–gene interactions for five RPL-associated genes (*AURKB, F2, F5, FOXD1,* and *PADI6*), with ribbons connecting each specific molecular action (e.g., “increases methylation,” “decreases expression”) to its respective gene target. Interaction data was obtained from merging the Intolerome and CTD datasets. BPA exhibited diverse regulatory interactions, especially increasing methylation and gene expression, while B[a]P mainly caused repressive changes, such as decreased methylation and reduced gene expression. Barplots (C) present Reactome pathway enrichment analyses of targeted genes with significant enrichment (FDR-adjusted p < 0.05) primarily in pathways related to hemostasis, including “Common Pathway of Fibrin Clot Formation,” “Formation of Fibrin Clot (Clotting Cascade),” and “Platelet Activation, Signaling, and Aggregation.” P-values were calculated using a hypergeometric test and adjusted for multiple comparisons using the Benjamini–Hochberg method.

For example, in Figure 5A, BPA appears to influence all five RPL genes through many unique interactions, like increasing or decreasing gene expression in *FOXD1* and *F5*, altering methylation states in *AURKB*, *PADI6*, and affecting protein abundance or enzymatic activity in *F2*. Similarly, Figure 5B shows that B[a]P modulates multiple genes, with observed effects including changes in methylation and suppression of expression across the subnetwork.

Panel 5C validates and extends these effects at the systems biology level. Both BPA and B[a]P exposures are enriched for disruptions in fibrin clot formation, coagulation cascades, and gamma-carboxylation of protein precursors. These biological pathways directly involve *F2* and *F5*.

The molecular interactions (as shown in Figure 5A and 5B) confirm that both BPA and B[a]P target the exact genes most enriched in our developmental lethality dataset, while the pathway enrichment (Figure 5C) reveals a shared downstream disruption of coagulation and implantation-related biology.

## Discussion

Our exposomic analysis identified associations between gene intolerance, developmental lethality, and environmental chemical exposures. Pairing chemical-gene interaction data also available in the Comparative Toxicogenomics Database (CTD) with Intolerome allowed the categorization of nearly 928 genes and ~4000 unique chemicals. Most of these genes were intolerant to loss-of-function (LoF) and were enriched in the metabolism, cardiovascular, and neurological pathways. Temporal concentration of lethality was in the late gestation and postnatal phases of development, indicating that variants in these genes may be observed during the development stages, i.e., physiological stress at immune maturation and organogenesis.

The majority of the chemicals involved in developmental lethality are annotated to fetal rather than maternal pathways (Figure 2A). This trend suggests that environmental exposures are more often connected to fetal-specific mechanisms, though it may also reflect the current emphasis of available studies and curated toxicogenomic data.

In profiling five RPL-associated genes, *F5, F2, AURKB, PADI6, and FOXD1*, we identified distinct exposomic network structures (Figure 3A–D, Figure 4). The exposomic profiles showed that *F5* and *AURKB* genes exhibit the highest node degrees, which could be caused by broad environmental sensitivity, while *PADI6* had a compact network with a narrower exposomic footprint. *FOXD1* and *F2* fell in between, with a moderate number of chemical connections. *FOXD1* regulates developmental patterning, while *F2* (thrombin) contributes to coagulation cascades; ultimately, both are integral to implantation and placental stability (National Center for Biotechnology Information, n.d.; Quintero-Ronderos et al., 2019). Narrowing down on each gene network provides a clear comparison of how different RPL genes are environmentally loaded, with *F5* and *AURKB* representing broad exposure profiles and *PADI6* reflecting sensitivity to a more limited but potent set of exposures.

Two chemicals, Bisphenol A (BPA) and Benzo[a]pyrene (B[a]P), interacted with all five genes, implicating shared toxicological mechanisms. Mechanistic annotations indicate that BPA and B[a]P modulate gene expression and methylation, particularly affecting pathways involved in coagulation and placental function. BPA is a widely used endocrine disruptor in polycarbonate plastics and thermal papers, resembling estrogenic action in breaking implantation, causing placental malfunction, and distorting fetal programming (Peretz et al., 2014; Susiarjo et al., 2013). Within the context of known biological effects (Figure 5A), BPA exposure causes transcriptome reprogramming and altered epigenetic marking phenotypes across the placenta and embryo (Cariati et al., 2020; Susiarjo et al., 2013). By contrast, B[a]P, a polycyclic aromatic hydrocarbon formed by incomplete combustion, largely works by activating the aryl hydrocarbon receptor (AhR), causing oxidative stress, formation of DNA adducts, and dysregulation of gene expression (Montano et al., 2025; Moorthy et al., 2002). Such effects can be explained by prior findings of B[a]P-driven chromatin rearrangement and transcriptional blocking in male gametes, which can interfere with embryonic development (Zhang et al., 2019). Both BPA and B[a]P have been associated with miscarriage, low birth weight, and other adverse pregnancy outcomes (Lathi et al., 2014; Muller et al., 2018; Zhao et al., 2024).

Molecular interactions (Figure 5A and 5B) show that both BPA and B[a]P target the same genes that are most abundant in our developmental lethality set, and pathway enrichment (Figure 5C) indicates that both activate downstream coagulation- and implantation-related biology. This is directly in line with our earlier results at the network level (Figure 4) and lethality timing (Figure 3), where *F5*, *F2*, and *AURKB* were the most interconnected and most vulnerable in the second trimester, a critical window for placental development. Thus, Figures 4 and 5 together not only confirm literature findings from toxicogenomic studies but also identify specific chemical pathways in genes that can influence RPL, suggesting a convergent mechanism of vascular or hemostatic compromise in early pregnancy failure

However, some notable limitations still persist. Firstly, incomplete or missing gene annotations, especially in the Intolerome database, full characterization of some genes and their roles in developmental disruption. Secondly, the CTD database does not account for exposure heterogeneity, emerging contaminants, or detailed exposure scenarios, which further limits its ability to model the actual conditions of the real world. Also, the lack of a particular population feature in our dataset, such as ancestry and environmental exposure context, limits the wider generalization of our results.

Finally, the absence of experimental validation limits mechanistic interpretation. To enhance translational potential and strengthen the biological relevance of our findings, future research should incorporate dynamic databases of chemical exposure, examine populations with diverse backgrounds, and employ experimental validation in model organisms.

Despite these constraints, our findings point to opportunities for clinical translation.CGA also has potential clinical utility in maternal–fetal medicine. Linking environmentally modifiable risks to intolerant genes can help contextualize the etiology of RPL and inform counseling. The same framework could be extended to related outcomes where chemical exposures are critical, including stillbirth and preterm birth.

Looking ahead, CGA offers a scalable and extensible framework for examining chemical-gene interactions beyond the specific case of RPL. It supports precision exposomics by allowing queries filtered by gene type (fetal or maternal), biological system, disruption type (loss- or gain-of-function), and timing of lethality. For example, users can identify placental genes expressed in the second trimester that are susceptible to environmental toxicants. Expanding CGA with additional datasets and real-world exposure profiles will further strengthen its value as a predictive toxicology and developmental risk assessment tool.

## Conclusion

This exposomic analysis reveals that genes with LoF intolerance, particularly those active in metabolic, cardiovascular, and neurological systems, are disproportionately impacted by environmental chemical exposures. By integrating CTD and Intolerome datasets, we created a curated map of 928 vulnerable genes and ~4000 chemical interactors, emphasizing genetic susceptibility during fetal and postnatal stages of development. The observed timing of lethality suggests latent, environment-driven disruptions during critical physiological transitions such as organogenesis and immune maturation. Exposomic profiling of five RPL-associated genes revealed conserved toxicant interactions, notably with Bisphenol A and Benzo[a]pyrene, which affected gene expression and methylation in coagulation and placental pathways. By integrating genomic intolerance and environmental interaction data, CGA offers a scalable and interactive tool to investigate and communicate clinically relevant, environmentally modifiable genetic risks.

## References

[R1] Almende B.V., & Thieurmel B. (2022). visNetwork: Network visualization using “vis.js” library (Version 2.1.2) [Computer software]. https://cran.r-project.org/web/packages/visNetwork/index.html

[R2] Bengtsson H. (2025). future: Unified parallel and distributed processing in R for everyone (Version 1.67.0) [Computer software]. https://cran.r-project.org/web/packages/future/index.html

[R3] Blengini C. S., & Schindler K. (2024). Genetic interaction mapping of Aurora protein kinases in mouse oocytes. Frontiers in Cell and Developmental Biology, 12, 1455280. 10.3389/fcell.2024.1455280 39386021 PMC11461192

[R4] Cariati F., Carbone L., Conforti A., Bagnulo F., Peluso S. R., Carotenuto C., Buonfantino C., Alviggi E., Alviggi C., & Strina I. (2020). Bisphenol A-Induced Epigenetic Changes and Its Effects on the Male Reproductive System. Frontiers in Endocrinology, 11, 453. 10.3389/fendo.2020.00453 32849263 PMC7406566

[R5] Cheng J. (2025). promises: Abstractions for promise-based asynchronous programming (Version 1.3.3) [Computer software]. https://cran.r-project.org/package=promises

[R6] Davis A. P., Wiegers T. C., Sciaky D., Barkalow F., Strong M., Wyatt B., Wiegers J., McMorran R., Abrar S., & Mattingly C. J. (2025). Comparative Toxicogenomics Database’s 20th anniversary: Update 2025. Nucleic Acids Research, 53(D1), D1328–D1334. 10.1093/nar/gkae883 39385618 PMC11701581

[R7] Fussell J. C., Jauniaux E., Smith R. B., & Burton G. J. (2024). Ambient air pollution and adverse birth outcomes: A review of underlying mechanisms. BJOG: An International Journal of Obstetrics & Gynaecology, 131(5), 538–550. 10.1111/1471-0528.17727 38037459 PMC7615717

[R8] Lathi R. B., Liebert C. A., Brookfield K. F., Taylor J. A., Vom Saal F. S., Fujimoto V. Y., & Baker V. L. (2014). Conjugated bisphenol A in maternal serum in relation to miscarriage risk. Fertility and Sterility, 102(1), 123–128. 10.1016/j.fertnstert.2014.03.024 24746738 PMC4711263

[R9] Montano L., Baldini G. M., Piscopo M., Liguori G., Lombardi R., Ricciardi M., Esposito G., Pinto G., Fontanarosa C., Spinelli M., Palmieri I., Sofia D., Brogna C., Carati C., Esposito M., Gallo P., Amoresano A., & Motta O. (2025). Polycyclic Aromatic Hydrocarbons (PAHs) in the Environment: Occupational Exposure, Health Risks and Fertility Implications. Toxics, 13(3), 151. 10.3390/toxics13030151 40137477 PMC11946043

[R10] Moorthy B. (1994). Chemical Structure- and Time-Dependent Effects of Polycyclic Aromatic Hydrocarbon-Type Inducers on Rat Liver Cytochrome P450, DNA Adducts, and I-Compounds. Fundamental and Applied Toxicology, 22(4), 549–560. 10.1006/faat.1994.1062 8056202

[R11] Müller J. E., Meyer N., Santamaria C. G., Schumacher A., Luque E. H., Zenclussen M. L., Rodriguez H. A., & Zenclussen A. C. (2018). Bisphenol A exposure during early pregnancy impairs uterine spiral artery remodeling and provokes intrauterine growth restriction in mice. Scientific Reports, 8(1), 9196. 10.1038/s41598-018-27575-y 29907759 PMC6003928

[R12] National Center for Biotechnology Information. (n.d.). F2—Coagulation factor II, thrombin (human) [Database record]. PubChem Gene. https://pubchem.ncbi.nlm.nih.gov/gene/F2/human

[R13] Peretz J., Vrooman L., Ricke W. A., Hunt P. A., Ehrlich S., Hauser R., Padmanabhan V., Taylor H. S., Swan S. H., VandeVoort C. A., & Flaws J. A. (2014). Bisphenol A and Reproductive Health: Update of Experimental and Human Evidence, 2007–2013. Environmental Health Perspectives, 122(8), 775–786. 10.1289/ehp.1307728 24896072 PMC4123031

[R14] Puche-Juarez M., Toledano J. M., Moreno-Fernandez J., Gálvez-Ontiveros Y., Rivas A., Diaz-Castro J., & Ochoa J. J. (2023). The Role of Endocrine Disrupting Chemicals in Gestation and Pregnancy Outcomes. Nutrients, 15(21), 4657. 10.3390/nu15214657 37960310 PMC10648368

[R15] Quenby S., Gallos I. D., Dhillon-Smith R. K., Podesek M., Stephenson M. D., Fisher J., Brosens J. J., Brewin J., Ramhorst R., Lucas E. S., McCoy R. C., Anderson R., Daher S., Regan L., Al-Memar M., Bourne T., MacIntyre D. A., Rai R., Christiansen O. B., … Coomarasamy A. (2021). Miscarriage matters: The epidemiological, physical, psychological, and economic costs of early pregnancy loss. The Lancet, 397(10285), 1658–1667. 10.1016/S0140-6736(21)00682-6 33915094

[R16] Quintero-Ronderos P., Jiménez K. M., Esteban-Pérez C., Ojeda D. A., Bello S., Fonseca D. J., Coronel M. A., Moreno-Ortiz H., Sierra-Díaz D. C., Lucena E., Barbaux S., Vaiman D., & Laissue P. (2019). FOXD1 mutations are related to repeated implantation failure, intra-uterine growth restriction and preeclampsia. Molecular Medicine, 25(1), 37. 10.1186/s10020-019-0104-3 31395028 PMC6688323

[R17] Intolerome. (2025). Intolerome gene list (Version 2025) [Data set]. University of California, San Francisco. https://rpldb.org/intolerome/

[R18] Sayal H. B., & Beksac M. S. (2024). The effect of hereditary thrombophilia on recurrent pregnancy loss: A retrospective cohort study. BMC Pregnancy and Childbirth, 24(1), 719. 10.1186/s12884-024-06926-w 39497077 PMC11536592

[R19] Susiarjo M., Sasson I., Mesaros C., & Bartolomei M. S. (2013). Bisphenol A Exposure Disrupts Genomic Imprinting in the Mouse. PLoS Genetics, 9(4), e1003401. 10.1371/journal.pgen.1003401 23593014 PMC3616904

[R20] Wu T., Hu E., Xu S., Chen M., Guo P., Dai Z., Feng T., Zhou L., Tang W., Zhan L., Fu X., Liu S., Bo X., & Yu G. (2021). clusterProfiler 4.0: A universal enrichment tool for interpreting omics data. The Innovation, 2(3), 100141. 10.1016/j.xinn.2021.100141 34557778 PMC8454663

[R21] Yu G., & He Q.-Y. (2016). ReactomePA: An R/Bioconductor package for reactome pathway analysis and visualization. Molecular BioSystems, 12(2), 477–479. 10.1039/C5MB00663E 26661513

[R22] Zhang W., Yang J., Lv Y., Li S., & Qiang M. (2019). Paternal benzo[a]pyrene exposure alters the sperm DNA methylation levels of imprinting genes in F0 generation mice and their unexposed F1–2 male offspring. Chemosphere, 228, 586–594. 10.1016/j.chemosphere.2019.04.092 31059956

[R23] Zhao N., Chu J., Liu J., Ma L., Ma N., Song W., & Sun T. (2024). Prenatal exposure to Benzo[a]pyrene affects maternal–fetal outcomes via placental apoptosis. Scientific Reports, 14(1), 17002. 10.1038/s41598-024-68029-y 39043924 PMC11266563

[R24] Zhou J., Mao R., Gao L., Wang M., Long R., Wang X., Li Z., Jin L., & Zhu L. (2024). Novel variants in PADI6 genes cause female infertility due to early embryo arrest. Journal of Assisted Reproduction and Genetics, 41(12), 3327–3336. 10.1007/s10815-024-03332-1 39644447 PMC11707103

[R25] Zinia S. S., Yang K.-H., Lee E. J., Lim M.-N., Kim J., Kim W. J., Ko-CHENS Study group, Park C., Kim H. J., Jung S.-W., Hong S., Jung A.-R., Lee J., Do Yu S., Hwang N., Jeong D. J., Seo H. W., Kim H. S., Lee M., … Sim C. S. (2023). Effects of heavy metal exposure during pregnancy on birth outcomes. Scientific Reports, 13(1), 18990. 10.1038/s41598-023-46271-0 37923810 PMC10624662

